# The Approach Behavior to Angry Words in Athletes—A Pilot Study

**DOI:** 10.3389/fnbeh.2019.00117

**Published:** 2019-06-04

**Authors:** Xue Xia, Jian Zhang, Xiaoshuang Wang, Xiaochun Wang

**Affiliations:** ^1^School of Psychology, Shanghai University of Sport, Shanghai, China; ^2^Faculty of Information Technology, University of Jyväskylä, Jyväskylä, Finland

**Keywords:** athletes, anger, aggressive behavior, behavioral approach system, theta oscillation

## Abstract

An increasing number of studies have found that athletes have a higher level of aggression than non-athletes. Anger is an important factor in the generation of aggressive behavior, and anger has been found to relate to both approach behavior and avoidance behavior. The present pilot study compared the aggression level of athletes and non-athletes using the Buss-Perry Aggression Questionnaire, and examined the responses of participants to anger-related stimuli using the manikin task, a paradigm that measures approach-avoidance behavior. In total, 15 athletes and 15 non-athletes finished the questionnaire and the manikin task, which included two conditions. In the anger approach condition, participants were asked to approach anger-associated words and to avoid neutral words. The instructions for the anger avoidance condition were the opposite (i.e., move away from the anger-associated words and toward the neutral words). Brain activity was recorded during the manikin task. Results showed that, compared with non-athletes, athletes had significantly higher physical aggression on the questionnaire. The athlete group showed significantly shorter reaction times in anger approach condition than anger avoidance condition. Theta oscillation activity induced during the anger approach condition was significantly lower than that during the anger avoidance condition in the athlete group. No significant correlation was found in present pilot study. These findings may suggest that when anger-related stimuli are present, athletes are more likely to approach, indicating stronger behavioral approach motivation that may result in aggressive behavior.

## Introduction

Human aggression is any behavior directed toward another individual that is carried out with the proximate (immediate) intent to cause harm. Also, the perpetrator must believe that the behavior will harm the target and that the target is motivated to avoid the behavior (Baron and Richardson, 1994; Anderson and Bushman, 2002). Several studies comparing aggressive behavior between athletes and non-athletes have found that athletes show a higher level of aggression than non-athletes (Rhea and Lantz, 2004; Rahimizadeh et al., 2011; Urzealǎ et al., 2014). However, less research has explored the related factors that may lead to the aggressive behavior of athletes.

The General Aggression Model which integrates multiple theories of aggression (Anderson and Bushman, 2002) highlights the importance of mood and emotion in aggressive behavior. Anger is an important emotion that reliably predicts self-reported aggression (Wyckoff, 2016). Otherwise, many studies have shown that aggressive behavior is associated with anger (Harmon-Jones and Sigelman, 2001; Harmon-Jones and Peterson, 2008; Harmon-Jones et al., 2010, 2013; Berkowitz, 2012). Thus, anger is a critical factor in aggressive behavior.

According to the motivational dimensional model of affect, each emotion, including anger, is associated with an approach or an inhibition motivational system, which is considered as the preparation for or inclination toward an action (Frijda et al., 1989; Lang, 1995; Bradley et al., 2001; Benvenuti et al., 2017). The behavioral approach system (BAS) is a motivational system that facilitates approach behavior, such as an attack, whereas the behavioral inhibition system (BIS) leads to avoidance and withdrawal motivation, such as an escape (Gray, 1990, 1994).

At present, some studies have revealed that anger-related information may trigger either approach or avoidance behavior (Blanchard and Blanchard, 2003; Marsh et al., 2005; Adams et al., 2006; Wilkowski and Meier, 2010; Mayan and Meiran, 2011; Bossuyt et al., 2014; Mcnaughton et al., 2016; Veenstra et al., 2017). On the one hand, compared with avoidance, it is easier to identify approaching anger stimuli or to complete an approaching task triggered by anger stimuli (Adams et al., 2006; Wilkowski and Meier, 2010). Higher trait anger predicts faster approach movements toward, rather than avoidance of, angry facial expressions, and makes it more likely that an approach will be selected in an approach-avoidance balance (Mayan and Meiran, 2011; Veenstra et al., 2017). On the other hand, anger has also been associated with avoidance (Blanchard and Blanchard, 2003; Marsh et al., 2005; Bossuyt et al., 2014; Mcnaughton et al., 2016). Using a manikin task, a study has found that when approach serves the goal to be aggressive and avoidance serves the goal to be submissive, anger is related to approach and fear is related to avoidance; however, when the goals are reversed, anger is then related to avoidance and fear is related to approach (Bossuyt et al., 2014). To date, however, no studies have revealed how the athletes reacted to anger-related stimuli in such circumstances.

Stimulus-response compatibility tasks, such as the joystick task (Fishbach and Shah, 2006), the feedback-joystick task (Rinck and Becker, 2007), and the manikin task (De Houwer et al., 2001; Krieglmeyer and Deutsch, 2010; Mogg et al., 2015), are considered as an effective paradigm for measuring motivational behavior. It has been observed that a desired stimulus immediately facilitates approach behavior, which is shown as a faster reaction to approach, whereas an undesired stimulus immediately facilitates avoidance behavior which is shown, through these tasks, as a faster reaction to avoid (Field et al., 2008; Zhou et al., 2012; Mogg et al., 2015). The manikin task requires participants to move a manikin toward or away from different kinds of stimuli. Since this task seems most sensitive to valence among these types of tasks (Krieglmeyer and Deutsch, 2010), it was adopted in the present study to reflect the motivational behavior of athletes and non-athletes to an anger-related stimulus.

Electrophysiological indexes, such as electroencephalography (EEG) activities, are important record indicators of the motivation system. Studies which monitored resting EEG brain wave patterns have found that left and right frontal brain activation represented by the alpha rhythm (8–13 Hz) are relevant to approach-related and avoidance-related emotions, respectively (Harmon-Jones and Allen, 1998; Harmon-Jones and Sigelman, 2001; Harmon-Jones, 2003; Carver and Harmon, 2009; Rodrigues et al., 2017; Prete et al., 2018). There is growing evidence that the midline posterior (Pz) vs. frontal (Fz) EEG theta activity (PFTA) serves as an effective index to reflect activation of approach motivation (Wacker et al., 2006, 2010; Walden et al., 2015; Reznik et al., 2017). PFTA, computed as the ln-transformed power at Pz minus the ln-transformed power at Fz, has been found to be positively correlated with self-reported levels of behavioral approach motivation measured by the Carver and White (1994) BIS/BAS scales (Wacker et al., 2010). However, both indexes are recorded during a relatively long period that includes the resting time or the middle of two trials, but they are not recorded throughout the entire task. By contrast, event-related theta oscillations (4–8 Hz) can be used to reflect the brain activity throughout a whole task (Moore et al., 2012; Mussel et al., 2016; Gheza et al., 2019). Event-related theta oscillations recorded throughout the whole task are believed to reflect the activity of brain systems that regulate behavior based on motivation-driven responses and mediate the association between an emotional stimulus and a behavioral response (Knyazev, 2007). Moreover, frontal midline theta is also related to negative emotion processing such as anger (Zhao et al., 2018), and it seems a better candidate than frontal alpha activity for use in a paradigm which is designed to modify emotional reactions (Mcfarland et al., 2017).

Therefore, the present pilot study aimed to determine whether athletes respond to anger-associated stimuli with approach behavior or with avoidance behavior. First, we measured the level of aggressive behavior in athletes and non-athletes using the Buss-Perry Aggression Questionnaire (BP-AQ), a personality questionnaire. Next, participants performed a manikin task while EEG recordings were obtained to compare the response to anger-associated stimuli and the associated brain activity in non-athletes vs. athletes. We hypothesized that compared with non-athletes, athletes would have higher aggression scores and a greater tendency to approach anger-related stimuli, as reflected by shorter reaction times (RTs) and lower event-related theta oscillations.

## Materials and Methods

### Participants

A total of 30 undergraduate students participated in the experiment, including 15 athletes as the athlete group and 15 non-athletes as the control group. The 15 athlete participants were all national second-level athletes with a mean sports experience of 9.7 years in basketball or football (eight males; mean age = 20.7, *SD* = 1.9). These two sports were chosen because the athletes in such events were widely regarded as more aggressive and more emotional (Uphill et al., 2014; Rui and Cruz, 2017; Cho et al., 2018). The 15 non-athletes had no regular sports experience and were age- and gender-matched to the athletes (eight males; mean age = 21.3, *SD* = 1.9). All participants were right-handed with normal or corrected-to-normal vision, from Shanghai University of Sport. The study was conducted in accordance with recommendations of the World Medical Association's Declaration of Helsinki and approved by the Shanghai University of Sport Ethics Committee (Shanghai, China). All participants gave informed consent to the study. After finishing the experiment, each participant was paid 50 RMB as compensation.

### Questionnaire

The Chinese version of State-Trait Anger Expression Inventory 2 (Spielberger, 1988; STAXI-II, Liu and Gao, 2012) was also adopted in the present study with the state anger scale (SAS), trait anger scale (TAS), and the anger expression scale (AX). The state anger changes over a short time span as it is related to the amount of anger experienced at a particular time. The SAS scale contains three subscales, named: (a) Feeling Angry (S_Ang_F); (b) Feel Like Expressing Anger Verbally (S_Ang_V); and (c) Feel Like Expressing Anger Physically (S_Ang_P). The trait anger is defined as a predisposition to experiencing anger, with two subscales: (a) Angry Temperament (T_Ang_T) and (b) Angry Reaction (T_Ang_R). The AX measures the expression and control of anger with four components: (a) Anger Expression-Out (AX-O); (b) Anger Expression–In (AX-I); (c) Anger Control–Out (AC-O); and (d) Anger Control–In (AC-I). The Cronbach α of internal consistency reliability was 0.243, 0.790, and 0.756, respectively, for SAS, TAS and AX, from previous unpublished data on 354 freshman undergraduates.

The BP-AQ (Buss and Perry, 1992) based on a five-point Likert scale with one representing “very often applies to me” and five representing “never or hardly ever applies to me” was used to assess the level of aggression. The Chinese version of BP-AQ which has been verified before was adopted in the present study (Fang, 2016). It contains 29 aggression-related statements with four subscales of aggression. Physical aggression (nine items) is a measure of hurting others physically. Verbal aggression (five items) measures the degree of hurting others verbally. The emotional or affective aspect of anger was measured by seven items. Hostility (eight items) measures the cognitive component of aggression (Buss and Perry, 1992). The BP-AQ has shown high internal consistency and good reliability for each subscale (Buss and Perry, 1992; Palmer and Thakordas, 2010). The Cronbach α of internal consistency reliability was 0.767 0.723, 0.81 and 0.814, respectively, for physical, verbal, anger, and hostility subscale, from previous unpublished data on 354 freshman undergraduates.

### Stimuli and Procedure

There were 110 stimuli words used in the study (50 anger-associated words, 50 neutral words, and 10 words were used for practice purposes only). Each word is a two-character word. We initially recruited 49 students (twenty-five males; mean age = 21.1, *SD* = 1.2) from the school who did not participate in the primary experiment. They were asked to rate the valence and arousal of the anger each word presented using a Likert scale, with anchors of 1 (not at all) and 9 (extremely). This process was done in the laboratory. The 200 words were chosen from the Chinese Affective Words System and included the most frequently used words with a familiarity degree of 5.29 ± 0.74 (Wang et al., 2008). Finally, the top 50 words were classified as anger-associated words (such as “betray,” “insult;” M_valence_ = 6.13 ± 0.22; M_arousal_ = 3.02 ± 0.19), and the lowest 50 words were classified as neutral words (such as “distribution,” “difference”; M_valence_ = 2.93 ± 0.15; M_arousal_ = 2.75 ± 0.31). These two types of words showed a significant difference in valence (*p* < 0.01).

**Figure 1:**
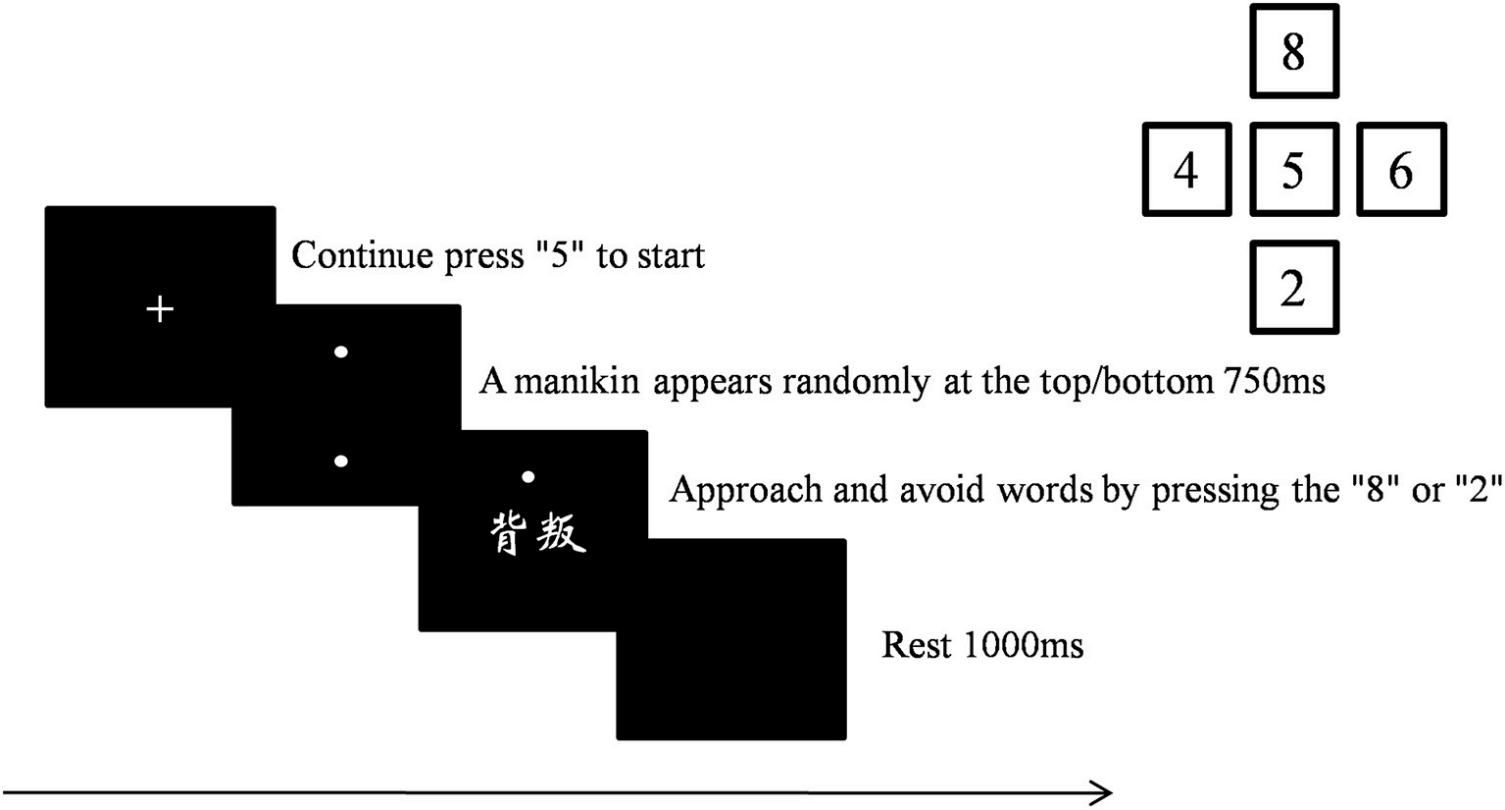
Manikin task procedure.

First, participants were asked to complete the STAXI-II and BP-AQ. Then, they were instructed to complete the manikin task, a stimulus-response compatibility task (De Houwer, 2003) modified based on De Houwer et al. (2001) and Krieglmeyer and Deutsch (2010). The manikin consisted of a circle for the head, a line for the body, and four lines, representing each arm and leg. The manikin was approximately 1.3 cm high and 0.9 cm wide. Participants could make the manikin move upwards by pressing the “8” key and could make it move downwards by pressing the “2” key with the right middle finger. First, a fixation point appeared in the center of the screen. Participants were instructed to press the “5” key with their middle finger continuously until pressing “2” or “8” to move the manikin. In this way, the middle finger always started from the same place to press other keys. After pressing “5,” the manikin appeared either in the upper or in the lower half of the screen with a 50% probability of each. After 750 ms, an anger-associated word or a neutral word was presented in the center of the screen. The task contained two conditions. For anger approach condition, participants were instructed to move the manikin toward anger-associated words and away from neutral words. For anger avoidance condition, the instructions were reversed (i.e., participants were asked to move the manikin toward neutral words and away from anger-associated words). The order of anger approach condition and anger avoidance condition was counterbalanced across participants. The task contained two blocks of 100 trials each, and the two types of words were presented an equal number of times. The RT was recorded between the word onset and the response. The manikin task procedure is illustrated in Figure 1.

### Behavioral Data Analysis

In accordance with a previous study (Mogg et al., 2015), RTs were excluded if they were <200 ms or more than 3 SDs above the mean (9% of data). Data from incorrect responses were also discarded (5% of data). The remaining behavioral data were imported into SPSS, version 20.0 and analyzed by repeated measures ANOVAs with the group (athlete and control) as a between-subjects factor and the condition (anger approach condition and anger avoidance condition) as a within-subject factor. *P*-value (two-tailed) < 0.05 was considered statistically significant. Greenhouse-Geisser corrections were used for within-subject factors and interactions when applicable.

### Electrophysiologic Data Acquisition and Analysis

EEG activity was recorded using the Brain Vision Recorder 2.0 system (Brain Products GmbH, Germany) with an electrode cap containing 64 sintered Ag-AgCl electrodes placed according to the International 10–20 system. The EEG data were referenced online against the FCz electrode and grounded at the AFz electrode. A vertical electrooculogram was obtained below the left eye, and the horizontal electrooculogram was obtained at the outer canthus of the right eye. The data sampling rate was 500 Hz, with 0.01–100 Hz band pass filtering by a Brain Amp amplifier. Electrode impedance was maintained below 5 k ohm during the experiment.

In general, two methods of time-frequency analysis are used with EEG data. In the first method, the data onto each single trial are analyzed, and then the average of the results of each single trial is determined in order to acquire phase-locked and non-phase-locked potential. This potential is the total activity contained in both the evoked and induced event-related oscillations (EROs). In the second method, data are averaged and then analyzed to acquire time-locked and phase-locked potentials called evoked EROs (Herrmann et al., 2004, 2014). The first method was adopted in the presented study. First, the reference electrode was converted offline to both posterior ear papillae, and the FCz electrode was restored using the Analyzer 2.0 system (Brain Products). The data were then pre-processed in MATLAB using the EEGLAB toolbox of Delorme and Makeig (Delorme and Makeig, 2004), and included the following steps: line noise was removed by a 50 Hz notch filter, followed by a high-pass filtering of 0.5 Hz and a low-pass filtering of 30 Hz, segmentation of the filtered continuous EEG into single trials (each trial was extracted offline from 950 ms pre-stimulus onset to 1,000 ms post-stimulus onset), baseline correction was achieved using the 950 ms preceding cue onset, and then artifact rejection was performed. A Wavelet filter was used and a complex Morlet continuous wavelet transform (CMCWT), based on the complex wavelet transform (Kronland-Martinet et al., 1987; Tallonbaudry et al., 1996; Demiralp et al., 2001), was used for time-frequency (TF) analysis of the EEG data in the MATLAB toolbox. CMCWT was described as below:

(1) $$\begin{array}{r} \text{CMCWT}\left(\text{t},\text{f}\right)=|\text{Φ}\left(\text{t},\text{ f}\right)*\text{x}\left(\text{t}\right){|}^{2} \end{array}$$

The time-frequency energy CMCWT (t, f) was used to calculate the convolution of the mother waveletΦ(t, f_c_)with the ERP data *x*(*t*) (Zhang et al., 2017). Here, Φ(*t, f*_*c*_) is the complex Morlet wavelet defined *as* below:

(2) $$\Phi \left(\text{t},{\text{f}}_{\text{c}}\right)=\frac{\text{1}}{\sqrt{\pi \sigma^{\text{2}}}}{\text{e}}^{\text{i2}\pi {\text{tf}}_{\text{c}}}{\text{e}}^{\frac{{\text{‒t}}^{\text{2}}}{\text{2}\sigma^{\text{2}}}}$$

In the above formula, f_c_ represents center frequency and σ represents bandwidth. A wavelet family was characterized by the constant ratio (Mørup et al., 2007):

(3) $$\text{K}=\;\frac{{\text{f}}_{\text{c}}}{\sigma_{\text{f}}}\text{ = 2}\pi \sigma {\text{f}}_{\text{c}}\text{ = 7}$$

For the Morlet, the half wavelet length was set to be 6 for the optimal resolutions of both frequency and time (Mørup et al., 2007; Cong et al., 2014). Then the time-frequency results of each single trial were averaged.

The appropriate electrodes and phase were selected based on the topographical distributions and time-frequency representations. Thus, the Fz, FCz, and Cz electrodes were selected for analysis of theta oscillation (4–8 Hz). Similar to the analysis of the behavioral data, repeated measures ANOVAs were used to analyze theta oscillations, with a group (athlete and control) as the between-subject factor and condition (anger approach condition and anger avoidance condition) as the within-subject factor.

## Results

### Self-Report Results

The scores of the STAXI-II showed no significant difference between two groups of each subscale as shown in Table 1. The results indicated that the level of state anger was consistent in both groups, and the trait anger was also comparable. The mean BP-AQ subscale scores and total scores of the two groups are shown in Table 2. *T*-tests found significant differences between the two groups in physical aggression, *t*_(1, 28)_ = −2.441, *p* = 0.021, and in total score, *t*_(1, 28)_ = −3.109, *p* = 0.004. There were no significant differences between groups in verbal aggression, anger or hostility scores.

**Table 1:** Descriptive statistics for the athlete group and control group of the state-trait anger expression inventory 2.

	**Control** (***N*** **=** **15)**		**Athlete** (***N*** **=** **15)**		***t***	***p***
	**Mean**	***SD***	**Mean**	***SD***		
Feeling Angry	5.47	0.92	5.40	0.74	0.220	0.828
Feel Like Expressing Anger Verbally	5.93	1.62	6.60	1.40	−1.203	0.239
Feel Like Expressing Anger Physically	5.20	0.41	5.47	0.52	−1.560	0.130
Angry Temperament	6.27	1.79	6.87	1.51	−0.993	0.329
Angry Reaction	10.73	2.94	12.00	2.36	−1.301	0.204
Anger Expression-Out	16.00	3.61	17.27	2.81	−1.072	0.293
Anger Expression–In	17.87	2.10	17.93	2.46	−0.080	0.937
Anger Control–Out	21.80	2.93	22.00	2.45	−0.203	0.841
Anger Control–I	23.40	2.35	22.60	2.67	0.871	0.391

**Table 2:** Descriptive statistics for the athlete group and control group of the buss-perry aggression questionnaire.

	**Control** (***N*** **=** **15)**		**Athlete** (***N*** **=** **15)**		***t***	***p***
	**Mean**	***SD***	**Mean**	***SD***		
Physical aggression	16.53^*^	3.78	19.87	3.70	−2.441	0.021
Verbal aggression	11.73	3.35	13.33	2.69	−1.443	0.160
Anger	14.87	2.67	17.87	5.24	−1.977	0.058
Hostility	16.80	6.73	20.20	4.90	−1.581	0.125
Total score	59.93^**^	9.93	71.47	10.38	−3.109	0.004

** p < 0.01;

* *p < 0.05*.

### Behavior Results

**Figure 2:**
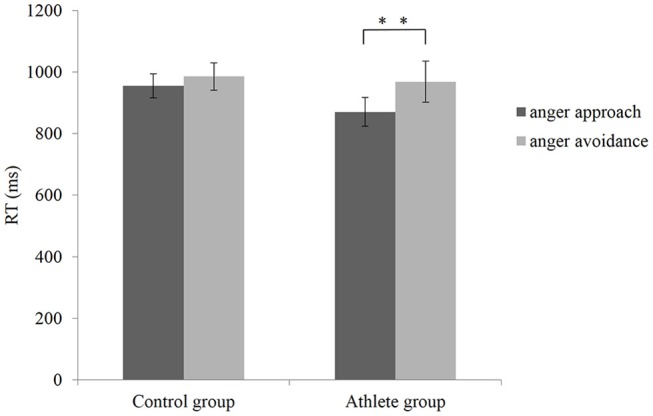
Mean RTs with SEM during anger approach condition (anger-approach/neutral-avoidance) and anger avoidance condition (anger-avoidance/neutral-approach) for the athlete group and the control group. ^**^p < 0.01.

A repeated measures ANOVA on RT in the manikin task showed a significant main effect of condition, *F*_(1, 28)_ = 15.831, *p* < 0.001, $\eta_{p}^{2}$ = 0.361, (1–β) = 0.970. Reaction time was faster in anger approach condition (mean = 912.870 ms, *SD* = 170.012) compared to anger avoidance condition (mean = 977.313 ms, *SD* = 216.237). The main effect of the group was not significant, *F*_(1, 28)_ = 0.537, *p* = 0.470, $\eta_{p\;}^{2}$ = 0.019, (1–β) = 0.109. A significant interaction was found between group and condition, *F*_(1, 28)_ = 4.322, *p* = 0.047, $\eta_{p}^{2}$ = 0.134, (1–β) = 0.519, as shown in Figure 2. The *t*-test showed that in the group of athletes, the RT of anger approach condition (mean = 870.573 ms, *SD* = 181.100) was shorter than anger avoidance condition (mean = 968.687 ms, *SD* = 259.270), within-group paired sample *t*-test: *t*_(14)_ = −3.379, *p* = 0.004, Cohen'd = 0.542. Whereas, there was no significant difference in the control group (within-group paired sample *t*-test: *t*_(14)_ = −2.143, *p* = 0.050, Cohen'd = 0.202; anger approach condition: mean = 955.167 ms, *SD* = 152.453; anger avoidance condition: mean = 985.940 ms, *SD* = 171.685). No significant difference between the athlete group and control group was found in both anger approach condition (independent sample *t*-test: *t*_(__28)_ = 1.384, *p* = 0.177, Cohen'd = 0.498) and anger avoidance condition (independent sample *t*-test: *t*_(14)_ = 0.215, *p* = 0.831, Cohen'd = 0.080).

### EEG Results

**Figure 3:**
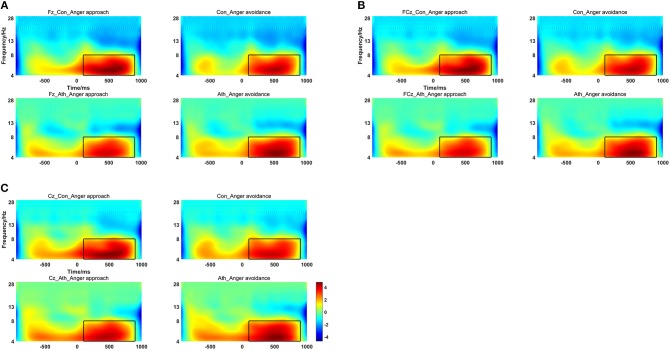
Time-Frequency Representations at the Fz, FCz and Cz electrodes, the time window of the rectangular area was from 100 to 900 ms and the frequency ranged from 4 to 8 Hz. **(A)** Fz electrode; **(B)** FCz electrode; **(C)** Cz electrode. Ath, athlete; Con, control.

**Figure 4:**
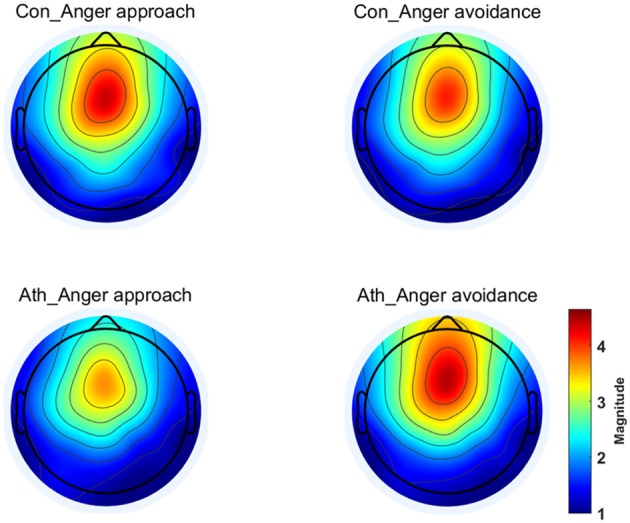
Topographical distribution of the theta oscillations at 100–900 ms. Ath, athlete; Con, control.

**Figure 5:**
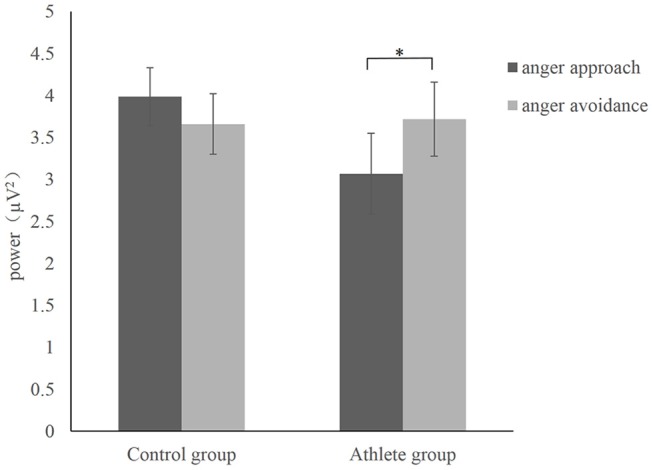
Mean power with SEM during anger approach condition (anger-approach/neutral-avoidance) and anger avoidance condition (anger-avoidance/neutral-approach) for the athlete group and the control group. ^*^p < 0.05.

The time-frequency representation of the two groups under the two conditions is illustrated in Figure 3. We chose 100–900 ms as the most active time window based on the time-frequency representations. The topographical distribution of theta oscillations (4–8 Hz) for the period of 100–900 ms is shown in Figure 4. A repeated measures ANOVA of theta oscillation power between 100 and 900 ms at the Fz, FCz and Cz electrodes showed that no significant main effect was found in the condition [*F*_(1, 28)_ = 0.597, *p* = 0.446,$\eta_{p}^{2}=$ 0.021, (1-β) = 0.116] and group [*F*_(1, 28)_ = 0.629, *p* = 0.434, $\eta_{p}^{2}=$ 0.022, (1-β) = 0.119]. There was a significant interaction effect between group and condition, *F*_(1, 28)_ = 5.516, *p* = 0.026, $\eta_{p}^{2}=$ 0.165, (1-β) = 0.621 (Table 3). The theta oscillation power induced by anger approach condition was significantly lower than theta power induced by anger avoidance condition in the athlete group (within-group paired sample *t*-test: *t*_(14)_ = –2.305, *p* = 0.037, Cohen'd = 0.349), not in the control group of non-athletes (within-group paired sample *t*-test: *t*_(14)_ = 1.071, *p* = 0.302, Cohen'd = 0.247) (Figure 5). No significant difference between groups was found in both anger approach condition (independent sample *t*-test: *t*_(28)_ = 1.556, *p* = 0.131, Cohen'd = 0.555) and anger avoidance condition (independent sample *t*-test: *t*_(28)_ = −0.104, *p* = 0.918, Cohen'd = 0.039).

**Table 3:** Theta power at the 100–900 ms time frame under anger approach condition and anger avoidance condition.

	**Athlete**		**Control**	
**Condition**	**Mean (μv^**2**^)**	***SD***	**Mean (μv^**2**^)**	***SD***
Anger approach condition	3.068	1.862	3.987	1.327
Anger avoidance condition	3.718	1.710	3.658	1.395

### Correlations Between Trait Aggression and Task Effects

The correlations between trait aggression and RT as well as the theta power are shown in Table 4. None of them passed Bonferroni correction (*P* = 0.0025).

**Table 4:** The correlation between the BP-AQ and the motivation index.

		**Physical aggression**	**Verbal aggression**	**Anger**	**Hostility**	**Total score**
RT-	*r*	−0.168	−0.134	−0.356	−0.305	−0.364
anger approach condition	*p*	0.375	0.480	0.054	0.101	0.048
RT–	*r*	0.011	0.042	−0.340	−0.106	−0.160
anger avoidance condition	*p*	0.955	0.824	0.066	0.578	0.398
Theta power–	*r*	−0.012	−0.044	0.125	0.034	0.014
anger approach condition	*p*	0.951	0.815	0.512	0.860	0.943
Theta power-	*r*	0.118	0.037	0.204	0.220	0.232
anger avoidance condition	*p*	0.534	0.845	0.280	0.242	0.217

*Bonferoni correction: P = 0.0025*.

## Discussion

This pilot study found that athletes showed higher levels of physical aggression as reflected by the BP-AQ and greater approach tendency to anger-related stimuli as reflected by shorter RTs and lower theta power in the manikin task.

For the self-report questionnaire, athletes scored significantly higher than non-athletes on physical aggression of the BP-AQ. However, there was no significant difference between groups in the Anger Expression-Out subscale which basically reflects aggression. This may be caused by the small sample size since, although not significant, the score of the athlete group is slightly higher than the control group. Thus, the present study indicated that athletes showed higher aggression than non-athletes, which may be caused by the excessive physical contact with other players in training and competition (Trivedi and Pinto, 2015; Sherrill and Bradel, 2017). But there is no doubt that we need to increase the sample size to consolidate this conclusion.

For the behavioral performance, the RTs of athletes were shorter in the anger approach condition than anger avoidance condition. Combining previous studies, anger-related information could trigger either both approach or avoidance behavior (Blanchard and Blanchard, 2003; Marsh et al., 2005; Adams et al., 2006; Wilkowski and Meier, 2010; Mayan and Meiran, 2011; Bossuyt et al., 2014; Mcnaughton et al., 2016; Veenstra et al., 2017); this finding may suggest that anger-related words induced athletes' stronger approach motivation than avoidance in present pilot study. Also, the simple effect analyses showed that a significant effect between conditions existed only in the group of athletes, not in the control group of non-athletes. This finding is similar to previous studies, in which people showed fast approaches to a positive stimulus (Krieglmeyer and Deutsch, 2010): smokers showed fast approach to a smoke-related stimulus (Mogg et al., 2015) and anorexia nervosa patients showed fast approach to low caloric food (Neimeijer et al., 2015). These results together may suggest that approach motivation sped up the approach behavior (Field et al., 2008; Krieglmeyer and Deutsch, 2010; Zhou et al., 2012; Mogg et al., 2015; Neimeijer et al., 2015). Thus, the behavioral results suggested that athletes showed a greater approach tendency toward anger-related stimuli.

At the neural level, theta oscillations were detected in all participants throughout the entirety of each condition. Frontal theta oscillation was associated with the motivational system (Knyazev, 2007, 2012) and emotional processing (Briggs and Martin, 2009; Walden et al., 2015; Benvenuti et al., 2017; Mcfarland et al., 2017; Zhao et al., 2018), and it could serve as a predictor of behavior, especially goal-directed behavior (Knyazev and Slobodskoj-Plusnin, 2007; Womelsdorf et al., 2010). Thus, the lower theta oscillation observed during the anger approach condition among athletes may suggest that approaching anger-associated stimulus was an act in which the task instruction (approaching) was in line with the motivational direction evoked by anger emotion, that is, the approach motivation rather than the avoid motivation. Frontal theta oscillation was also closely related to cognitive effort and to the frontal control which related to response inhibition effort (Kirmizi-Alsan et al., 2006; Wacker et al., 2006; Hanslmayr et al., 2008; Cavanagh et al., 2009). A previous study found that, after high provocation, enhanced theta activity was observed during the trials with no aggressive reaction. Instead, reduced theta was observed in trials with more aggressive behavior, which indicated that reduced theta activity reflected the low cognitive effort and response inhibition (Krämer et al., 2009). Consistent with previous study, lower theta oscillation in the anger approach condition than in the anger avoidance condition among the athlete group may suggest that less cognitive effort was needed to approach anger-associated stimuli. Combining the behavioral results with the EEG results, the RT along with the lower theta oscillations in the anger approach condition suggested that an anger-related approach was easier for athletes when compared with avoidance. However, it should be noted that the observed power of most statistical results was very low which indicated that more data based on a larger sample size would be needed to confirm these ideas.

We did not find a significant correlation between aggression and motivation in the present pilot study. Previous study has found that occipital theta oscillation was associated with the processing of angry facial expressions; more specifically, elevated theta was observed for angry faces compared to happy faces (Diao et al., 2017). However, no significant correlation about the theta oscillation and the anger or motivation processing was found in the present study, which may suggest that more evidence would be needed for explaining the relationship between the theta oscillation and motivation.

## Limitations and Future Directions

This study has some crucial limitations that bear consideration when interpreting our findings. First, the sample size was small, which limited the statistical power and meant all of the results were underpowered, especially for group comparisons. Second, emotion related variables were not well controlled; for instance, we did not measure the participants' emotions to confirm they were angry or nor and we did not directly induce anger. This meant we could only investigate the reaction to anger-related words, not the impact of anger itself. Also, no other emotional stimuli were included in the present study, so we cannot confirm that the approaching effect was specific to anger, or all other emotions. Thus, future study should exert an emotional induced paradigm to investigate the impact of emotions such as anger, fear, happiness, or others on the motivational behavior. Additionally, non-contact athletes should also be included in the future study to gain deeper insight, as the significant difference was found in the level of aggression between contact athletes and non-contact athletes (Boostani, 2012; Trivedi and Pinto, 2015).

## Conclusions

Despite these limitations, the present pilot study revealed that athletes showed a higher level of physical aggression and an approach tendency toward anger-associated stimuli at both behavioral and neural levels, which suggested that an approach tendency associated with anger-related information may be an important factor in triggering aggressive behavior among athletes. However, these findings should be cautiously interpreted until studies with larger samples and stronger power are able to replicate the findings.

## Ethics Statement

The study was conducted in accordance with recommendations of the World Medical Association's Declaration of Helsinki and approved by the Shanghai University of Sport Ethics Committee (Shanghai, China).

## Author Contributions

XcW, XX, and JZ designed experiments and conducted experiments. XsW and XX analyzed data. XX and XcW wrote the paper.

### Conflict of Interest Statement

The authors declare that the research was conducted in the absence of any commercial or financial relationships that could be construed as a potential conflict of interest.
